# A Survey to Assess Family Physicians’ Motivation to Teach Undergraduates in Their Practices

**DOI:** 10.1371/journal.pone.0045846

**Published:** 2012-09-28

**Authors:** Marcus May, Peter Mand, Frank Biertz, Eva Hummers-Pradier, Carsten Kruschinski

**Affiliations:** 1 Institute of Clinical Pharmacology, Hannover Medical School, Hannover, Germany; 2 Institute of General Practice, Hannover Medical School, Hannover, Germany; 3 Institute of Biometry, Hannover Medical School, Hannover, Germany; 4 Department of General Practice/Family Medicine, Georg-August University, Goettingen, Germany; Pennington Biomedical Research Center, United States of America

## Abstract

**Background:**

In Germany, family physicians (FPs) are increasingly needed to participate in undergraduate medical education. Knowledge of FPs’ motivation to teach medical students in their practices is lacking.

**Purpose:**

To describe a novel questionnaire that assesses the motivation of FPs to teach undergraduates in their practices and to show the results of a subsequent survey using this instrument.

**Methods:**

The questionnaire was developed based on a review of the literature. Previously used empirical instruments assessing occupational values and motivation were included. A preliminary version was pretested in a pilot study. The resulting 68-item questionnaire was sent to 691 FPs involved in undergraduate medical education. Reliability was assessed and subgroups were analyzed with regard to differences in motivation.

**Results:**

A total of 523 physicians in n = 458 teaching practices participated (response rate 75.7%). ‘Helping others’ and ‘interest’ were revealed as the predominant motives. Responses showed a predominantly intrinsic motivation of the participating FPs. Their main incentives were an ambition to work as a medical preceptor, to generally improve undergraduate education and to share knowledge. Material compensation was of minor importance. Time restraints were indicated as a barrier by some FPs, but were not a general concern.

**Conclusion:**

German FPs involved in medical education have altruistic attitudes towards teaching medical students in their practices. Motivational features give an important insight for the recruitment of FP preceptors as well as for their training in instructional methods.

## Introduction

Family physicians (FPs) are commonly involved in undergraduate teaching in Germany as well as worldwide. In German medical schools, there is a compulsory 1- to 3-week full time practical training/clerkship in certified peripheral family practices, the so-called ‘teaching practices’. These clerkships usually take place in the more advanced study years, often in the 3^rd^ clinical year, usually after the other major clinical rotations. In comparison to Anglo-Saxon countries these clerkships are rather short. [1]–3. FP preceptorships in Germany are based on one-on-one instruction, FPs are expected to introduce medical students to the characteristics of practice-based family medicine, e.g. the gate-keeper role or long-term chronic care. As part of these clerkships, students should interview and examine patients on their own. However, teaching in the practices is not very standardized, as in Germany, teach-the-teacher programs are not generally implemented. However, many Medical Faculties/Institutes of Family Medicine have developed guidelines for their academic teaching practices. Studies have shown that teaching in ambulant care settings is beneficial [4], [5] and even valued as advantageous compared to medical education in the hospital. [6], [7] Current modifications to medical education in Germany generally demand a higher amount of practical training for undergraduates. [1], [8] As a consequence, there is a much greater need for physicians with involvement in medical education, including ‘one-on-one’ teaching in general practices. [9].

Specific training in instructional methods for physicians involved in medical education is known to be beneficial and is currently implemented in German faculty development programs. [10] However, teaching is very time-consuming and poorly remunerated compared to patient care. [11]–[14] Considering these disincentives, physician’s motivation and their satisfaction of teaching students in their practices need to be very high to make a good preceptor (a practising physician giving practical training to a medical student). [15] Enthusiasm for teaching is a known characteristic of medical educators and should be a basic qualification for physicians who want to participate in medical education. [16] Considering the decreasing numbers of FPs [17] it is even more important to recruit highly motivated physicians to serve as role models and to increase the proportion of graduates entering family practice. [18].

In Germany, knowledge regarding FPs’ motivation to teach is lacking and specific instruments are rare. The aim of this study was to assess the motivation of German FPs involved in medical education. Therefore, a comprehensive motivational questionnaire was developed based mainly on previously published instruments. The motivational aspects prompting FPs to start teaching medical students, to continue teaching, or barriers encountered were assessed.

## Methods

**Figure 1:**
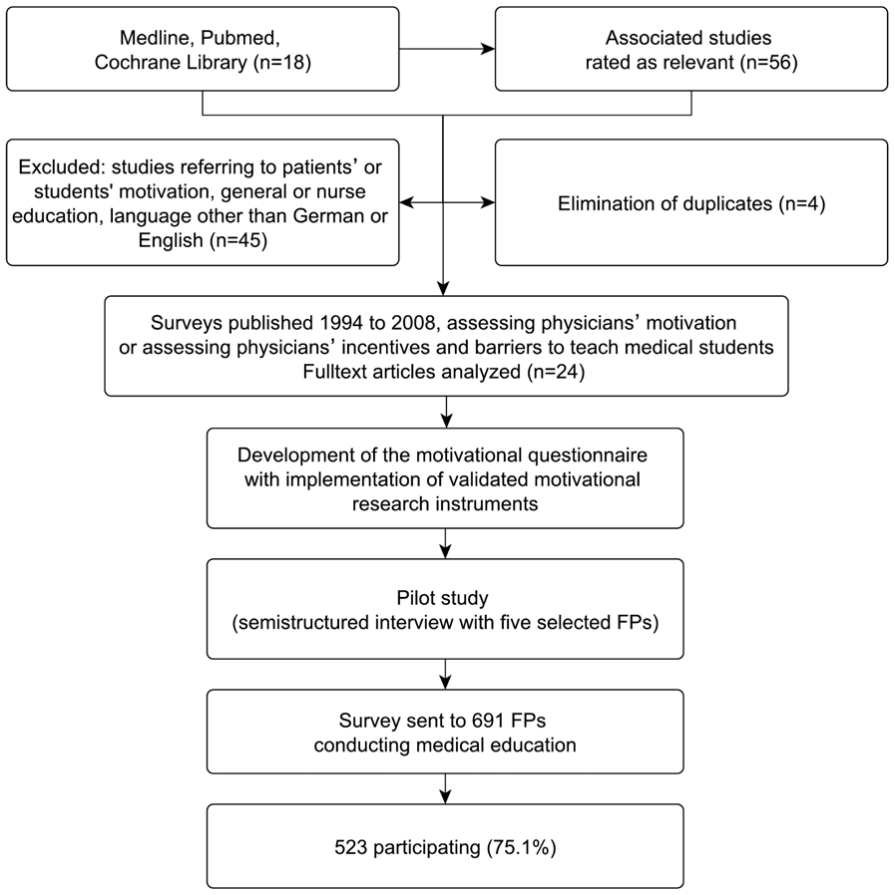
Flow-Chart.

Figure 1 gives an overview of the study design.

### Questionnaire Development

First, a systematic literature review was conducted to reflect all relevant incentives to start and to continue teaching, as well as benefits and barriers for teaching students in family practice settings. The extensive literature review comprised a search for validated instruments assessing motivational factors to teach medical students.

Literature searches included Pubmed, Medline and Cochrane Library databases using the MeSH-Terms ‘Education, (medical)’; ‘Motivation’; ‘Satisfaction, (personal)’; ‘Teaching’; and ‘Physicians’. Each abstract was studied and the corresponding papers were ordered if considered relevant. Additional studies were identified by citation checking of the reference lists of studies initially identified. Inclusion criteria were surveys published from 1994 to 2008 assessing physicians’ motivation or physicians’ incentives and barriers to teach medical students. Exclusion criteria were languages other than German or English, studies referring to patients’ or students’ motivation, general or nursing education, or to other topics than physician’s motivation. Opinion papers, comments, and Editorials were not considered relevant. Following an assessment of quality, e.g. checking for obvious methodology deficits or surveys with insufficient or insufficiently explained methodology, some pieces of empirical research were excluded, as well. All studies that were considered relevant (n = 24) are summarized in table 1.

**Table 1:** Literature review.

Ref.	Authors	Journal	Main results
[49]	Baldor RA et al	Medical Education; 2001	High interest in teaching regardless of discipline, practice structure, payment or teaching experience; hindrances: increased workload, median extended working hours per day (60 minutes); positive effects: being up-to-date, more job satisfaction; rewards: awards and special concessions are valued as important, payment is important for half of the responders
[32]	Dahlstrom, J. et al.	BMC medical education; 2005	Important factors: altruism, intellectual satisfaction, improve their own skills and seek the truth, negative: little involvement in course structure, too much work, wasted time
[26]	Dodson, M. C.	Obstetrics and gynecology; 1998	At least moderate interest, negative impact on patient flow, seminars or conferences to improve teaching skills, potential rewards discounts on computers, sporting and cultural events, books, support with educational material
[47]	Fulkerson, P. K.; Wang-Cheng, R	Family medicine; 1997	Most important is personal satisfaction (84%), appropriate rewards: University affiliation, discounts, workshops
[33]	Gerrity, M. S et al.	Journal of general internal medicine; 1997	Joy of teaching, interaction with students, expectations: participation in study structure, relief, monetary compensation
[11]	Grayson, M. S.; Klein, M.; Lugo, J.;Visintainer, P.	Journal of general internal medicine; 1998	Better job satisfaction through teaching, training through apprenticeships, kept up to date through contact with students, held in higher esteem by patients; negative: decline in number of patients
[12]	Hartley, S.; Macfarlane, F.; Gantley, M.; Murray, E.	BMJ (Clinicalresearch ed.); 1999	Positive effect through professional support, improvement of teaching skills, improving clinical skills and knowledge, broadening horizons, contact with enthusiastic students, better image problems: lack of space and time, concerns about lack of patient care while teaching
[34]	Hill, N.; Wolf, K. N.; Bossetti, B.; Saddam, A.	Journal of allied health; 1999	Most rewarding aspect is observing student’s development, low student motivation and poor personal and professional behavior of students is frustrating, benefits of teaching reduced by increased pressure due to restructuring of health care
[52]	Hoban, J. et al.	Academic medicine; 1996	Incentives: personal enrichment, recognition/reward, little interest in personal benefits, incentive and teaching program required
[50]	Kumar, A.; Kallen, D. J.; Mathew, T.	Teaching and learning inmedicine; 2002	Personal satisfaction highest score, in contrast payment, gifts or services judged less important, some emphasis on rebates and awards
[35]	Kumar, A. et al.	Journal of general internal medicine; 1999	Payment between 13% and 22%, education 70% to 89%, 90% to 95%, academic awards, special events 62% to 79%, thank you letters 74% to 84%, minimal difference between disciplines
[48]	Langlois, J. P.	Family medicine; 1995	Payment, financial aid and textbooks are possible incentives for participation, different weighting of the answers
[31]	Latessa, R et al.	Family medicine; 2008	High degree of satisfaction with teaching, negative impact on patient flow, increase of working-time due to teaching, interest to promote general medicine, family doctors material rewards valued higher rather than awards, less satisfied with income
[36]	Latessa, R. et al.	Academic medicine; 2007	High level of satisfaction with teaching 93.0%, planed to continue teaching for the next five years 90.9%, satisfied with incentives 57.2%, physicians report a negative impact of teaching students on their professional life
[13]	Levy, B. T.; Gjerde, C. L.; Albrecht, L. A.	Academic medicine; 1997	More time in practice because of teaching 87%, fewer patients seen 31%, loss of practice income 25%, highest interest in training and access to computer-based information, motivation for teaching is the positive interaction with the students and the satisfaction of teaching, lack of time is the most difficult aspect
[44]	Rutter, H.; Herzberg, J.; Paice, E.	Medical education; 2002	No evidence of more stress by teaching, even signs of stress reduction
[39]	Scott, I.; Sazegar, P.	Medicalteacher; 2006	Main reason is joy in teaching, main hindrances are lack of educational skills and too high a workload
[53]	Single, P. B.; Jaffe, A.; Schwartz, R.	Family medicine; 1999	Decision to participate in the medical course of study dependent on past experience and influence of students on patient care, continuation of medical education was stated as primary incentive, financial incentives the least important; no homogeneous responses, reward of CME points preferred to financial remuneration.
[37]	Starr, S. et al.	Academic medicine; 2003	Feeling of satisfaction most often, knowledge and ability to teach such as member of a group of teachers is strengthening the role as a teacher, being a physician means being a teacher, responsibility to teach, sharing ones experience, only a few interested in receiving payment, some would appreciate acknowledgement from the university
[38]	Starr, S. et al.	Teaching and learning in medicine; 2006	Satisfaction of teaching, knowledge and abilities to teach, belonging to a community of teachers, receiving awards for teaching, being a physician means being a teacher, responsibility to teach, sharing experiences, aspects of ‘Teacher Identity’
[40]	Ullian, J. A.; Shore, W. B.; First, L. R.	Academic medicine; 2001	Special skills not required, possible rewards for continuing medical education as recognition and appreciation, material rewards e.g. reduced fees for library; joy in teaching primary motive for participation, benefits outweigh the disadvantages (patient flow and income decreased, more work, etc.)
[41]	Vath, B. E.; Schneeweiss, R.; Scott, C. S.	The Western journal of medicine; 2001	Workload increased 63%, reduced time for teaching 56%, joy of teaching students is the most important factor
[14]	Vinson, D. C.; Paden, C.	Academic medicine; 1994	Increase in time at work (average 46 minutes per day)
[23]	Wright, S. M.; Beasley, B. W.	Mayo Clinic proceedings.Mayo Clinic; 2004	Physicians with stake in medical education value ‘helping others’ as main incentive compared to scientifically active physicians who value self-expression more highly

Only a few validated instruments applicable into a short motivational survey were available. [19] Informed by our literature review, two motivational instruments, the Rosenberg Occupational Values Scale (ROVS) [20] and the Questionnaire of Current Motivation (QCM, original validated German version) [21], were considered best suited to the particular question and adapted to the current survey. Both instruments have been used previously in motivational surveys. [22]–[25].

The ROVS is a frequently used tool to evaluate person’s job specific motives. Morris Rosenberg developed the original ROVS in 1957 to assess students’ career motivation. The scale is based on ten items referring to ‘people-oriented’, ‘extrinsic reward-oriented’ and ‘self-expression-oriented’ value domains. [20] Rosenberg’s assumptions of intrinsic and extrinsic motivation shaping career choice form the basis of many motivational research articles, but the items have always been modified according to the specific situations. [22], [23] The Scale was translated into German and adapted culturally. Finally, 13 items based on the ROVS and the studies from Wright et al. [23] and Dodson et al. [26] were used to characterize the ongoing factors that motivate FPs to teach.

Resulting(current) motivation depends on ongoing motivation as well as occupational conditions. [27] Therefore, after a few minor adaptations the QCM was added in total to the survey to assess factors of current motivation and thus enhance the motivational questionnaire. As a pilot, to check whether there were obvious differences in Germany and to test if the questionnaire was broadly applicable, five FPs that had successfully participated in undergraduate education were selected and interviewed to assess both the questionnaire and the feasibility of the survey (pilot study). Feedback was used to slightly adapt the questionnaire specifically for German physicians as well as to increase the clarity of the instrument.

The final questionnaire consists of a total of 68 items. Practice demographics, as well as location and practice structure are assessed with 9 items to clarify family physicians’ working environment in Germany and to evaluate possible correlations to motivational findings. The next 41 items are statements gathered from the literature research in order to reflect personal and situational motives. Physicians are asked to rate their agreement/disagreement (5-point Likert scale: ‘strongly disagree’ ( = 1) to ‘strongly agree’ ( = 5)) with statements related to incentives to start teaching (7 items), and to continue teaching (12 items), as well as benefits (11 items in total, 8 advantages for the physician and 3 for the student) and difficulties (11 items) with teaching students in their own practices. The 13 items based on the ROVS are implemented in these 41 statements assessing the three ongoing motives ‘helping others’, ‘self-expression’, and ‘extrinsic rewards’. Finally, the 18 items of the QCM follow, also using the 5-point Likert scale (‘strongly disagree’ ( = 1) to ‘strongly agree’ ( = 5)). From their analysis, the four factors ‘probability of success’, ‘providing a challenge’, ‘interest’ and ‘fear of failure’ arise and characterize the current motivation.

### Survey

Each of the n = 691 FPs that regularly offered practice-based teaching in the Universities of Göttingen (n = 122), Hannover (n = 169), Magdeburg (n = 242) and Ulm (n = 158) were invited by mail to participate in the survey with the questionnaire. To increase the response rate, physicians were reminded of the survey after two weeks.

### Statistical Analysis

Statistical analysis consisted of basic measures for assessing demographics. Means were calculated for incentives, benefits, and barriers. Sum scores were determined for the different aspects of motivation assessed by our instrument (‘helping others’, ‘self-expression’, ‘extrinsic rewards’ and ‘probability of success’, ‘providing a challenge’, ‘interest’ and ‘fear of failure’). Within the ‘five-point Likert scale’ a score of three was defined as a neutral response [28]. Using factor analysis and calculation of Cronbach’s alpha, reliability of the survey was analyzed [29]. Theoretically, alpha varies from zero to 1, with higher values indicating a higher reliability but also often a high redundancy. The degree of reliability considered appropriate depends upon the use of the instrument. In instruments intentionally designed to be as short as possible (as in the current survey) usually a somewhat lower reliability is accepted.

In Germany, institutional review board approval is not needed for surveys with voluntary participation and no collection of personal data. Data were stored and analyzed in an anonymous form.

## Results

### Sociodemographic Details of the Participating FPs (Table 2)

**Table 2:** Characteristics of participating FPs (n = 523).

Gender male, n (%)/female, n (%)/missing n (%)	349(66.7)/172(32.9)/2(0.4)	
Age (years), mean ± SD [min-max, median]	53.8±7.7 [36–74, 54]	
Time own practice (years), mean ± SD [min-max, median]	19.2±8.7 [2]–[49,19]	
Time conducting student’s education (years), mean ± SD [min-max, median]	5.8±3.9 [2]–[34,4]	
Solo practice, n (%)	277(53.0)	
Group practice (%)	186(35.6)	
Practice-sharing (%)	41 (7.8)	
Ambulatory healthcare center (%)	3(0.6)	
Authorization for postgraduate education yes (%)/no(%)	363(69.41)/160(30.59)	
vocational trainee in past five years yes (%)/no(%)	222(42.4)/299(57.2)	

After the first invitation to participate, 425/691 physicians responded (61.5%). Following the reminder, the total increased to 523/691, to give a final response rate of 75.7%. The mean age was 54 years, 19 years in private practice and six years of involvement in medical education. FPs were mainly male (66.7%). The proportion of the participants working in rural practices was 49.7%. The majority of the FPs (53%) practiced single-handed, the others worked in joint practices (35.6%) or in group practices (8.4%). Of the participating practices, 69.4% were accredited for post-graduate education (vocational training), and in the preceding five years 57.2% of these had hosted a post-graduate, vocational trainee. In Germany, physicians need to be accredited for postgraduate education by the chamber of physicians.

### Incentives to Start Teaching

Table 3 depicts incentives, benefits and barriers with regard to becoming a preceptor (delivering practical training to undergraduate medical students in one’s own practice, full time for 1 to 3 weeks). Contact/affiliation to the local university ranked highest as initial motivating factor. Advertizing from the university and payment were rated as less important.

**Table 3:** Incentives to start and maintain teaching; benefits and barriers in teaching (5-point Likert scale from ‘strongly disagree’ = 1 to ‘strongly agree’ = 5).

		Mean(SD)	1(%)	2(%)	3(%)	4(%)	5(%)
Motivation for taking-up	Affiliation/Contact to University?	**3.25**(1.33)	16,15	12,12	20,96	32,31	18,46
a medical training	Targeted Recruitment	**2.87**(1.67)	37,69	7,50	10,38	18,65	25,77
position	Seminar/Course	**2.64**(1.28)	26,97	17,73	26,97	20,62	7,71
	Contacts/Recommendation	**2.49**(1.64)	47,28	10,89	8,56	12,45	20,82
	Unspecified Recognition	**2.43**(1.24)	31,08	22,01	25,48	15,25	6,18
	Title of ‘Academic Trainer’?	**2.14**(1.16)	41,43	19,46	24,86	11,75	2,50
	Payment	**1.87**(1.06)	50,58	21,43	20,46	5,02	2,51
Motivation to continue	Pass on family physician knowledge	**4.36**(0.82)	1,34	2,50	6,53	38,58	51,06
	Improve medical teaching	**4.24**(0.96)	2,30	3,84	11,52	32,44	49,90
	Interest in promoting family medicine	**4.16**(1.04)	3,26	5,18	12,28	30,52	48,75
	Interested to take on the responsibility of teaching	**3.97**(0.96)	1,93	5,59	19,08	40,85	32,56
	Experience from your own training	**3.88**(1.09)	4,03	7,68	19,39	34,17	34,74
	Is a professional expectation (Hippocratic oath)	**3.86**(1.08)	3,84	8,06	19,19	35,89	33,01
	The challenge to keep current	**3.77**(1.03)	3,84	7,10	22,07	41,84	25,14
	To refresh medical knowledge	**3.59**(1.07)	5,37	9,21	26,10	39,92	19,39
	Contact to the University	**3.06**(1.13)	10,36	20,15	32,63	27,06	9,79
	To overcome professional isolation	**2.88**(1.34)	22,46	15,55	26,10	23,03	12,86
	To find a successor	**2.11**(1.29)	45,58	22,31	14,81	9,81	7,50
	Family or practice tradition	**1.70**(1.16)	65,45	14,20	9,60	5,95	4,80
Advantages of teaching	To present the complexity of family practice	**4.69**(0.66)	1,16	0,58	2,13	20,16	75,97
students	To convey experiences	**4.59**(0.71)	1,36	0,78	2,91	26,94	68,02
	To impart an understanding of patient	**4.39**(0.76)	1,17	0,97	6,80	39,81	51,26
	Abilities and social competencies	**3.33**(1.10)	7,74	12,19	32,69	33,85	13,54
	Personal satisfaction	**3.19**(1.10)	8,74	15,53	34,95	29,71	11,07
	Patients are pleased with medical students	**3.10**(0.95)	5,43	19,19	40,70	29,26	5,43
	It’s positive for care	**2.88**(1.06)	11,46	22,72	37,48	22,91	5,44
	Supports care activities	**2.43**(1.05)	19,77	36,82	26,74	13,57	3,10
	Higher profile	**2.28**(1.03)	27,22	31,47	29,54	10,04	1,74
	Important for the future	**2.19**(1.05)	31,91	30,56	25,34	10,83	1,35
	Increased income	**1.49**(0.83)	66,15	23,15	7,59	1,56	1,56
Disadvantages of	Lack of time for teaching	**3.05**(1.13)	10,85	19,96	31,40	28,68	9,11
teaching students	Students with insufficient background knowledge	**2.86**(1.12)	14,42	21,25	34,11	24,37	5,85
	Performance measurements	**2.77**(1.23)	18,48	25,49	25,68	21,21	9,14
	Insufficient time available to refresh knowledge	**2.57**(1.06)	18,83	27,57	33,98	16,89	2,72
	Interruption of routines	**2.48**(1.11)	22,03	31,38	27,49	15,01	4,09
	Insufficient knowledge of teaching techniques	**2.33**(1.03)	25,63	30,49	31,07	10,87	1,94
	Insufficient pay	**2.19**(1.26)	40,66	23,74	18,09	10,89	6,61
	Insufficient recognition	**2.16**(1.16)	38,45	25,24	22,91	8,93	4,47
	Burden	**2.08**(0.99)	34,50	32,36	24,56	7,60	0,97
	Unmotivated disinterested students	**1.81**(0.97)	47,47	32,10	14,59	3,50	2,33
	Direct and indirect supervision	**1.80**(0.93)	47,28	32,30	14,79	4,47	1,17

### Incentives to Continue Teaching

Among the incentives to continue teaching, the ‘interest to transfer knowledge’ was most highly valued by the FPs, closely followed by the ‘desire to improve medical education’, ‘to promote family medicine’, ‘to take responsibility for teaching’, ‘to apply one’s experience in education’, ‘appraisal as an occupational duty’, ‘to stay up to date’ and ‘to update medical knowledge’. It was less important either to ‘continue the tradition’, ‘find a successor’ or ‘to overcome isolation’.

### Benefits of Teaching for Physicians

The benefit of teaching students in the FP practice was dominated by ‘allowing students to get a better idea of the work undertaken by a family physician’, followed closely by ‘presenting personal experiences’ and ‘better understanding of frequent patient concerns’. There was strong disagreement with the statement that involvement in teaching may have a positive effect on practice income. Equally, ‘benefits for the future’, ‘higher prestige’, ‘support by the students’ and ‘better patient care’ seemed to be less important factors.

### Barriers for Teaching

The only disadvantage identified as a relevant barrier for teaching according to the 5-point Likert scale, was a ‘lack of time’, which was relevant for some FPs, but not of a major concern. The other barriers had a mean value of less than three.

### Results of the ROVS and the QCM (Table 4)

**Table 4:** Motivational subscales of the ROVS and QCM.

Scale	Subscales	Item	Mean±SD	Factor analysis	Score±SD	Cronbach Alpha
**ROVS**	**Self expression**	Challenges/motivation of the students	3.77±1.02	0.624	3.14±0.74	0.654
		Proof of social competency	3.33±1.10	0.812		
		Personal satisfaction	3.19±1.10	0.828		
		Increased esteem from patients	2.28±1.03	0.506		
	**Helping others**	Interest in promoting family medicine	4.16±1.04	0.564	4.46±0.62	0.766
		Understanding of frequently asked patient questions	4.39±0.76	0.843		
		Appreciation of the complexities of familymedicine	4.69±0.66	0.894		
		Passing on personal experiences	4.59±0.71	0.857		
	**Rewards**	Support of supply activities	2.43±1.05	0.621	2.42±0.67	0.704
		Useful for my future	2.19±1.05	0.665		
		Patients enjoy contact with students	3.10±0.95	0.762		
		Improved patient treatments	2.88±1.06	0.811		
		Improved income	1.49±0.82	0.495		
**QCM**	**Probability of success**	To be equal to problems	4.26±0.77	0.519	3.4±0.64	0.359
		Likelihood of failure	3.04±1.20	0.772		
		Anyone can be a preceptor	2.64±1.19	–0.093		
		Belief in failure	2.36±1.17	0.841		
	**Providing a challenge**	Correct challanges	3.46±1.06	0.751	3.47±0.74	0.652
		Anticipation of success	2.85±1.21	0.770		
		Determination	3.93±0.93	0.638		
		Pride in success	3.65±1.01	0.630		
	**Interest**	I like teaching	4.37±0.81	0.793	3.61±0.67	0.633
		Identification as a teaching Doctor	4.01±0.82	0.757		
		Interesting tasks	3.97±0.87	0.735		
		No payment necessary	3.08±1.30	0.424		
		Also in my free time	2.61±1.34	0.552		
	**Fear of failure**	Under pressure	1.79±0.94	0.698	1.74±0.66	0.633
		Fear of blame	1.66±0.88	0.775		
		Embarrassment of failure	1.89±1.08	0.722		
		Concern about requirements	2.00±0.99	0.774		
		Paralyzed by the demands	1.38±0.74	0.581		

Ongoing motives for physician involvement in teaching, determined by the ROVS, were clearly dominated by ‘helping others’. ’Self-expression’ emerged as moderate and ‘extrinsic rewards’ as much less important. Actual motivation, determined by the QCM, resulted in nearly the same values for ‘interest’, ‘challenge’, and ‘probability for success’, whereas ‘fear of failure’ was rated lower (Table 4), a pattern of motivation indicating a high positive motivation. [30].

Factor analysis and Cronbach’s alpha were calculated for the subscales of the ROVS and the QCM. Within the subscale ‘probability of success’ factor analysis revealed ‘everyone can be a preceptor’ as a probable misconception in adaption to the current survey. Disregarding this, calculated alphas were between 0.63 and 0.77, indicating an acceptable reliability of the instruments (Table 4).

## Discussion

A questionnaire was developed and applied to determine FPs’ motivation to teach undergraduate medical students in their practices. In general, the participating physicians were highly motivated by the ambition to help others and the actual motivation was dominated by the genuine interest to teach students. The participating doctors showed a selfless, altruistic attitude towards teaching which confirmed earlier physicians’ motivation results [23], [31]. However, there are some striking differences with our work presented here. In our study, ‘promoting family medicine’ was rated as very important. According to the applied Likert Scale, 78.3% of the physicians rated this item with approval (data of relative frequencies not shown). Yet, elsewhere a percentage of only 33% of the participating FPs found this aspect important [31].

The evaluation of the ROVS and QCM supports the theory that doctors enjoy teaching and that they are motivated by the desire to provide students with a good education. Motives in regard to ‘Helping others’ were generally scored high, whereas motives in regard to ‘Extrinsic rewards’ and ‘Self-expression’ were of minor importance for the FPs in our study. These findings are consistent with previous studies examining physicians’ incentives to teach [23], [26], [32]. One earlier study reported that more than half of the participating physicians mentioned at least a moderate interest in teaching [26], and other studies have suggested that the satisfaction in teaching was the most important reason for their participation in medical education [13], [31], [33]–[41]. Factors for the resulting (actual) motivation, revealed by the QCM, were dominated by interest in teaching, which was closely followed by challenge and probability of success. According to the literature these special intrinsic motivational characteristics - recording high values for an interest in the work itself accompanied by moderate results in challenge, a good probability of success, and a low anxiety - indicate a high motivation and reflect an autonomous type of self-directed responsibility. [30], [42] Moreover, this specific motivational pattern is supposed to reduce stress at work and ensure a highly motivated state called ‘flow’ [43]. This may be the reason why physicians who teach students in their practices have less stress at work even though they have an increased workload [44].

The most important motives for the FPs in our study to work as a preceptor were to improve medical education, promote family medicine and to give the students a good education. Other studies have revealed that a lack of perceived teaching skills is an important barrier for occasional teaching (teaching periods alternate with time without students). [39] This was the case in our group of physicians. Our findings might however be confounded by the fact that teaching is voluntary for German Family Practices, and that practices accredited for postgraduate training may be overrepresented, since these are preferentially recruited as teaching practices. This implies that FPs have significant experience and a profound interest in teaching.

In Germany, students seem to regard the field of work of other specialties as more prestigious than FP’s work. [45] The chance to improve the image of FPs through higher representation of Family Medicine within Universities [46] might be an important reason for physicians to contribute to in medical education. In line with this assumption and results of other studies [47], the predominant incentive to become involved in medical education seems to be an affiliation with or contact to the host university. Accordingly, advertizing letters from the university appear to represent a practical and well accepted means to recruit further physicians.

In contrast to our results, other studies have found that payment or other rewards in exchange for FP teaching contributions have been found to be important [48], [49]. Disparities might depend on the amount of the payment, which even the German lay press has commented to be very low, or may reflect the design of the questionnaire used in the present study. Physicians in our study were not asked directly if they considered remuneration important. Instead when queried in relation to their motivation to teach medical students in their practices, payment was rated as not relevant. Interestingly, in one of the studies by Kumar et al. the payment was valued more highly than awards or privileges [50]. However, in another earlier study also conducted by Kumar et al. [35], intangible rewards were considered much more important than payment. Presumably, though the amount of recompense is less important, being paid at all might be considered as the universities token of respect or approval of FP teaching and therefore still be important for their motivation.

Previous studies have revealed several disadvantages associated with teaching medical students in private practices (Table 1). Most of these disadvantages are related to the various educational or health care systems and, therefore, do not seem to be generally applicable in Germany. The fact that teaching might influence practice workflow (and income) negatively is consistent with previous results. Other observations have revealed a negative impact on daily working time of nearly one hour per day [13], [14], [49]. These data fit well to the results of the current survey with regard to barriers for teaching in the physician’s own practice. Insufficient time was identified as the only relevant adverse motivational factor.

The reliability and congruence of responses to the instruments used in this paper were comparable to other well-established instruments to assess job satisfaction [51]. Only one item, that ‘everyone can be a preceptor’ was shown by Cronbach’s alpha and factor analysis to not fit to the subscale ‘probability of success’. Perhaps this item should be reworded or deleted in subsequent research. For now, it remains unclear whether the motivation measured might contribute to the high quality of teaching. The teaching evaluation results for n = 134 of the participating physicians given by the attending students at the family practice training were available. The results varied from very good to excellent, with a very small standard deviation (data not shown). Correlations between motivation and teaching skills could not be determined.

Altogether, our findings support the idea that offering students a good education seems to be the central motivation among physicians with participation in the medical course of study and that the main prerequisite for medical preceptors is a high intrinsic motivation. [16] The doctors in our study seem to teach for the sheer love of teaching, which is known as a rare, favorable and worthwhile property of good medical preceptors. [52] In Germany, the amount of private practice teaching in Family Medicine settings is generally low. The compulsory Family Medicine clerkship lasts up to three weeks, but in many faculties it is organized as a one-week-clerkship only. Recent reform plans [1] suggest a minimum of two weeks, which might be obligatory for all faculties in the future. Moreover, it is discussed to implement an additional three-month Family Medicine clerkship as compulsory part of the final practical year (4^th^ clinical year). As described in the introduction, our study anticipated these plans, as many more academic teaching practices will be needed in this case. The initial enthusiasm of the FPs involved in our study may regress in a few years. Thus, it is very important to create incentives for the FPs to strengthen their motivation to teach. Revealed by our current survey intrinsic motivation is the dominating factor of the physicians’ motivation to teach. This doesn’t mean that extrinsic rewards like remuneration are not necessary in the future. As stated above, even if the physician did not indicate the remuneration as important for their motivation, many might decline to participate without payment. One option to strengthen the physicians’ motivation might be through their increased participation in the medical curriculum development. This would likely reinforce their feeling of being ‘self-determined’ preceptors. With respect to the FPs ambition to improve medical education and promote family medicine, such involvement would probably represent an appropriate reward for them.

### Conclusion

The questionnaire developed and reported here serves as a tool to ascertain and clarify the motivation of FP’s towards teaching medical students in their practices. Motivational research is based on determining reasons why people choose, start, and maintain actions [27]. These principles were applied in the survey. According to the current results, a high interest in teaching students and helping others as an important ongoing motive were revealed as fundamental characteristics of FPs with good teaching skills. The applicability of the questionnaire should be further investigated. Furthermore motivational findings should be compared to FPs without involvement in medical education to investigate whether the pattern of motivation found here is generic to German FPs, or specific to those who opted to engage in teaching.
